# Gonadorelins adherence in prostate cancer: A time‐series analysis of England’s national prescriptions during the COVID‐19 pandemic (from Jan 2019 to Oct 2020)

**DOI:** 10.1002/bco2.101

**Published:** 2021-08-19

**Authors:** Ravina Barrett, Robert Barrett, Kalyan Dhar, Brian Birch

**Affiliations:** ^1^ School of Pharmacy and Biomolecular Sciences Cockcroft Building University of Brighton Brighton UK; ^2^ School of Pharmacy and Biomedical Sciences University of Portsmouth Portsmouth UK; ^3^ Portsmouth UK; ^4^ Department of Gynaecological Oncology Swansea Bay University Health Board Singleton Hospital Swansea UK; ^5^ University Hospital Southampton NHS Foundation Trust Southampton UK; ^6^ School of Medicine University of Southampton Southampton UK

**Keywords:** COVID‐19, follow‐up, gonadotropin‐releasing hormone, hormonal therapy, male, prostate cancer

## Abstract

**Objective:**

To examine the effect of the COVID‐19 pandemic on gonadorelin analogue prescription for community patients in England.

**Materials and methods:**

We included data from all primary‐care patients who had relevant prescriptions dispensed in the community in England. Descriptive statistics and interrupted time series analysis over 22 months (15 months before and 7 months after lockdown) was evaluated.

**Results:**

A total of 22 months’ worth of data were analyzed (or 1 041 638 total items, monthly average 47 347 items). Goserelin; leuprorelin, and triptorelin are the medicines most used by total quantity in the study period. Simple descriptive statistics show that mean values have declined during the pandemic. The Interrupted Time Series (ARIMA Modeling) shows declining trends.

After the pandemic's onset, we observe a statistically significant downward trend for goserelin (*P* = .017) and leuprorelin (*P* = .014). As these are the major constituents of the model, we interpret this overall data as showing a significant downward category trend. Aside from linearity, a significant step change was noted for leuprorelin (*P* = .029) showing an increase in prescription items with a similar effect that is close to being statistically significant for goserelin (*P* = .051).

The actual cost of medicines shows minimal variation suggesting that prices of individual medicines have remained stable. The regional data showed variation but this was not statistically significant. In all cases, the Oct‐20 figures are lower “year on year.” This novel work reports the impact of a global pandemic on prescription volumes of prostate cancer (PCa) medicines.

**Conclusions:**

A worrying decrease in prescription medicines raises concerns for the care of PCa patients. We encourage diagnosed patients to discuss their planned care with their doctor.

## 1 INTRODUCTION

Cancer is a leading cause of death worldwide, accounting for an estimated 9.6 million deaths in 2018.^1^ Prostate cancer (PCa) is the second most common cancer affecting men worldwide.^2^, ^3^ Risks include advanced age, ethnicity, obesity, and family history.^4^ Globally, PCa is common (1.28 million cases), where approximately 70% of deaths from cancer occur in low‐ and middle‐income countries.^1^ In the UK, PCa continues to be the most common cancer diagnosed in males in 2017 (41 201 cases) accounting for one in four (26.3%) of male cancer diagnoses.^5^ The “Cancer Prevention and Control” through an Integrated Approach and 2030 UN Agenda for Sustainable Development^6^, ^7^ seek to reduce premature mortality from such cancers.

Prostate cancer is usually slow‐growing and often asymptomatic at diagnosis.^8^ The presenting symptoms of advanced disease include urinary outflow obstruction, renal failure, and pain due to bone metastases.^9^ Treatment decisions are guided by baseline prostate‐specific antigen (PSA) levels, tumor grade (Gleason score), tumor stage, patient life expectancy, treatment morbidity, and patient preference.^9^

Androgen deprivation therapy (ADT) is often used in the treatment of advanced PCa due to its effect in lowering testosterone to castrate levels via the hypothalamic‐pituitary‐gonadal axis (HPA). Gonadotrophin‐releasing hormone (GnRH) analogues are synthetic agents that mimic the actions of luteinizing hormone releasing hormone in the body.

Administration of GnRH analogues (but not antagonists) produces an initial phase of hormone stimulation. With continued administration, there is down‐regulation of GnRH receptors, thereby reducing the release of gonadotrophins (follicle stimulating hormone and luteinizing hormone) which in turn leads to inhibition of androgen production in men. This effect is reversible on GnRH analogue discontinuation. In men, around 21 days after the first injection, testosterone concentrations fall to within a “castrate range” (ideally <0.4 nmol/L) and remain suppressed with continuous treatment, usually given by injection every 28‐84 days. This inhibition usually leads to PCa regression and symptomatic improvement. In metastatic PCa, these agents show similar outcomes to surgical castration.^10^ They can also be used as short‐term adjuvants^11^ with radiotherapy for up to 2 years in the treatment of localized PCa. In women, GnRH analogues have been used in hormone‐dependent advanced breast cancer, uterine fibroids,^12^ endometriosis,^13^ and suppression of follicular development within the ovary, where they cause endometrial thinning and amenorrhea^14^. In children they may be used to address growth and maturation deficiencies.^15^, ^16^ As a result, these medications have a wide spectrum of use,^17^, ^18^ though clinically they are overwhelmingly used for the treatment of PCa.^19^

The PCa treatment is intended to relieve symptoms, achieve disease remission and improve overall quality of life. From a public health, primary care perspective, it is important that these (potentially elderly) patients continue to get their medicines at timely regular intervals to ensure disease progression is delayed as long as feasibly possible. Due to the variable recovery of the HPA, natural testosterone levels recover slowly and so in patients exhibiting a good response to treatment discontinuation of therapy (intermittent ADT) is feasible. However, at some time patients usually stop responding to ADT and experience rising PSA levels despite castrate levels of serum testosterone (castrate resistant PCa or castrate‐resistant prostate cancer).^20^ However, due to the variable recovery of the HPA, natural testosterone levels sometimes never recover and discontinuation of therapy (intermittent ADT) is feasible. For younger patients, if timely administration is missed, rising testosterone levels may cause further disease progression.^21^, ^22^ Generally, cancer sufferers are also at higher risk of contracting infections (eg LUTS, COVID‐19), as a consequence of the cancer itself or the medication they take to treat it.

Triptorelin (Decapeptyl® and Gonapeptyl®), leuprorelin (Prostap®), and goserelin (Zoladex®) are GnRH analogues licensed for use monthly, 3‐monthly, or 6‐monthly in the form of prefilled syringes and vials. National Institute for Health and Care Excellence (NICE) guidelines^23^ recommend that men with PCa should be followed up in primary care according to locally agreed protocols. For men with advanced disease guidelines suggest the use of GnRH analogues if PSA levels are above locally agreed thresholds or if there is symptomatic progression. In normal clinical practice, we expect to see patients attending the GP surgery at monthly, 3‐monthly or 6‐monthly intervals for “top up” injections to control disease as evidenced by a low PSA. As a result, there should be minimal variation in prescription volumes.

## 2 AIMS AND OBJECTIVES

This study hypothesizes that the COVID‐19 pandemic would not affect community prescription issuance of GnRH analogues and that prescription volumes would be relatively stable during the first wave of the pandemic in England.

## 3 METHODS

The “English Prescribing Dataset” (EPD)^20^ contains public sector information licensed under the Open Government Licence V2.0, which provides anonymized prescription data in England.^24^ The EPD contains detailed information on community‐issued prescriptions (not hospital) issued in England but which were dispensed across the UK (England, Wales, Scotland, Guernsey, Alderney, Jersey, and the Isle of Man). It contains detailed prescribing information at practice level, which are aggregated by British National Formulary (BNF) code (eg, 0803042P0AAACAC for “Triptorelin 11.25 mg inj vials”) to maintain patient confidentiality. Therefore, each row of data is aggregated at practice level and does not represent individual patients or prescriptions. The data include total quantity of unit‐doses (eg, pre‐filled syringes, vials), and “actual cost” for reimbursement.

The data exclude prescriptions issued outside England (Wales, Scotland, Guernsey, Alderney, Jersey, and the Isle of Man—this difference is solely to do with where prescriptions were issued as opposed to where dispensed); items not dispensed, disallowed, and those returned for further clarification; prescriptions prescribed and dispensed in prisons, hospitals, and private prescriptions; items prescribed but not presented for dispensing or not submitted to NHS prescription services by the dispenser. This dataset includes small operational irregularities (eg, 17 rows in Jan 2019 of “unidentified practice data,” 470 rows of “NULL” chemical substance codes where accurate BNF codes were given to permit extraction of the missing data). The study population represents English residents who were issued a prescription and had it dispensed.

Monthly data from January 2019 to October 2020 were examined for all potential PCa medicines and included (Goserelin Acetate [0803042K0], Leuprorelin Acetate [0803042N0], Triptorelin [Acetate] [0803042P0], and Triptorelin Embonate [0803042S0]). We excluded Triptorelin Embonate (0607020Y0) and Histrelin Acetate (0803042Q0) because they are archived or “special” codes that are not routinely used or last used over 5 years ago. Note that Triptorelin Embonate was included (0803042S0) and excluded (0607020Y0) as per specific codes.

Approximately 387 288 884 rows of data (528.8 gigabytes of data) were extracted using Structured Query Language (SQL). After excluding unnecessary rows, the data were filtered down to 12 092 relevant rows of data. Twenty‐two comma‐separated values (CSV) files were imported into a Microsoft SQL® server table labeled EPD. As each one was imported, it was validated and assigned an exact data type (eg, “Total quantity” is a “floating” data point, “regional office name” is a textfield) to each field of data. The data were cleaned by the removal of spaces, blanks, and checked for incorrect data formatting (eg, that text characters were not in a numeric field or vice versa). Microsoft Visual Studio® was used to create and edit SQL Server Integration Services® (SSIS) packages that imported, validated, and consolidated the data within an automated import routine, (see Supplemental Methods [Data Cleaning]) for details. Data were aggregated by month, chemical substance, regional office name, and BNF code, to allow for human analysis. The CSV data allow for comparison across months. Detailed population analyses were not conducted and were assumed to be constant. The target population cohorts consisted of patients prescribed and dispensed these medicines, but their exact diagnosis remains undocumented and therefore unknown. Lockdown commenced on March 23, 2020 with subsequent local and national lockdowns.

## 4 ANALYSIS

Analysis was carried out in Excel® v. 2007 and SPSS® v. 26. Results are presented as proportions, descriptive statistics, and hypothesis testing at 95% confidence level and by monetary value in pounds sterling (government provided).

Interrupted time series (ITS) analysis was used to fit time trends.^25^ A commonly used time series modeling framework (autoregressive integrated moving average or ARIMA) was employed to analyze the monthly total‐quantity of prescription data from the EPD. ARIMA is a flexible modeling construct, allowing lagged correlations and seasonal differences to be modeled, but we initially used only a simple model with no allowance for serial correlation nor seasonality, mainly due to the lack of data points after the interrupt time point. We had available 22 consecutive monthly data points with the interrupt time set at the 14th month (March 2020), and 15 data points before and seven data points after March 2020. We estimated the difference in prescription total‐quantity as at March 2020, and also the difference in the linear trend (ie, between the slopes of the lines) before and after the interrupt time point. We then added an autoregressive component (lag of one month) to make some allowance for short‐term serial correlation. The observed temporal trend in prescription total‐quantity was explored visually in advance of performing the main time series analysis. See Supplemental Methods (Sensitivity analysis, Syntax).

Medicine sample validation was done against https://openprescribing.net/. While every effort has been made to validate the data, it has not been possible to independently validate against manufacturers. We checked the supply status and made enquiries with the manufacturer^26^ regarding the supply volumes of Zoladex® 3.6 mg (PL 17901/0064); Zoladex LA® 10.8 mg (PL 17901/0065) Implant because they have been used for over 30 years and are a major data constituent.

Ethical approval was not required for this database study and no patient identifiable data were accessed. There was no public and patient involvement.

## 5 RESULTS

A total of 22 months’ worth of data were analyzed (or 1 041 638 total items, monthly average 47 347 items), using March 2020 as the cut‐point for the first lock‐down in England, making it nationally representative.

### 5.1 By total quantities of medicines

Goserelin acetate; Leuprorelin acetate; Triptorelin (Acetate) and Triptorelin embonate are the medicines most used by total quantity in the study period (see Figure S1 and Supplemental Results [Table S1]). We present price‐data in Supplemental Results (Table S2). Regional analysis is presented in Supplemental Results (Figure S1, Table S3). See also detailed individual formulations analysis on Total Quantity and Actual Costs in Supplemental Results (Table S4, Table S5).

Simple descriptive statistics of the 15 months before the pandemic and the 7 months after its onset are presented in Table 1, which shows that mean values have declined during the pandemic except for Triptorelin embonate which is proportionately, a smaller constituent. Given its relative lack of use, its exclusion was considered, but is presented here for completeness. Percentage changes are also noted in the final column of Table 1, for potential population of 47 000 patients.

**TABLE 1:** Descriptive statistics for the monthly total quantities of prescribed medicines items‐standard deviation (STD), 95% confidence intervals (CI)

Medicines		Months, N	Mean	STD	95% CI	Before/after % change
Goserelin acetate	Before pandemic	15	19 884	842	(20 309;19 459)	
	Lockdown onward	7	19 455	972	(20 176;18 734)	−2%
Leuprorelin acetate	Before pandemic	15	17 933	772	(18 323;17 543)	
	Lockdown onward	7	17 631	1005	(18 376;16 886)	−2%
Triptorelin (Acetate)	Before pandemic	15	7040	350	(7216;6864)	
	Lockdown onward	7	6964	319	(7199;6729)	−1%
Triptorelin embonate	Before pandemic	15	906	40	(926;886)	
	Lockdown onward	7	1100	93	(1169;1031)	+21%

### 5.2 Interrupted Time Series (ARIMA Modeling)

**FIGURE 1:**
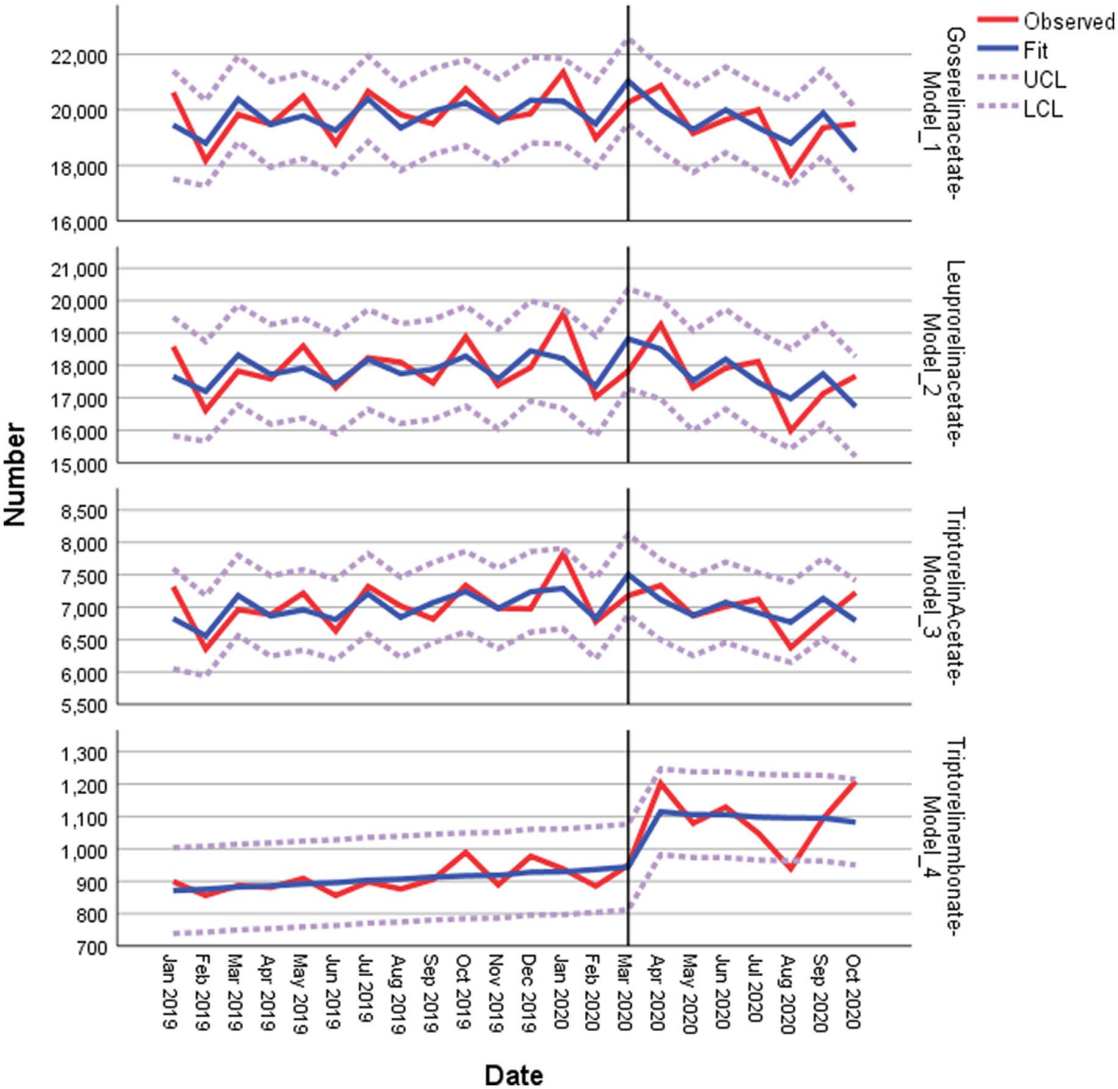
Auto regressive integrated moving average (ARIMA) model—with 1‐month autocorrelation (1,0,0) (0,0,0)

Since prescription data are not random, a 1‐month autocorrelation better reflects routine clinical practice. The model allows for correlation with the previous month's prescription volumes (see Table 2 and Figure 1).

**TABLE 2:** ARIMA (1,0,0)(0,0,0) Model Parameters—Estimated change in prescription volumes at March 2020, Confidence intervals (CI); T‐statistic (T, Stat); standard error (SE)

	Parameter estimate	SE	T, Stat	*P*‐value	95% CI
*Estimated slope BEFORE March 2020*					
Goserelin acetate	59	29	2.04	.058	(−1;119)
Leuprorelin acetate	39	30	1.30	.211	(−23;102)
Triptorelin (Acetate)	30	12	2.55	.**021**	(6;54)
Triptorelin embonate	5	4	1.39	.183	(−2;13)
*Estimated slope AFTER March 2020*					
Goserelin acetate	−270	102	−2.64	.**017**	(−482; −58)
Leuprorelin acetate	−292	106	−2.76	.**014**	(−513; −72)
Triptorelin (Acetate)	−79	42	−1.90	.075	(−165;7)
Triptorelin embonate	−10	13	−0.79	.443	(−38;17)
*Post‐ vs Pre‐effect (step‐change)*					
Goserelin acetate	4000	1909	2.10	.051	(40;7960)
Leuprorelin acetate	4735	1981	2.39	.**029**	(627;8843)
Triptorelin (Acetate)	1067	777	1.37	.187	(−544;2678)
Triptorelin embonate	335	244	1.37	.189	(−172;841)

Table 2 suggests that steadily growing monthly parameter estimates of the injectables before the pandemic go into accelerated reversal after March 2020, but are accompanied with a positive step change. In supplemental sensitivity analysis, we find significant percentage changes for each medicine within our model using a natural logarithm transformation.^27^, ^28^ We estimate the percentage changes as an autoregressive function at 1 month lag, however, this should be interpreted with caution.^29^

Before the pandemic, Triptorelin acetate (*P* = .021) was showing evidence of increasing prescription volumes, which is captured in the significant linear trend for prescription statistics before March 2020.

After the pandemic's onset, we observe a statistically significant downward trend for goserelin (*P* = .017) and leuprorelin (*P* = .014) accompanied with much lower confidence intervals. As these are major constituents of the model, we interpret this overall data as showing a significant downward category trend.

Aside from linearity, we also examined whether there was a step change. A significant step change was noted for Leuprorelin (*P* = .029) showing a jump in prescription items with a further jump that is close to being statistically significant for goserelin (*P* = .051). Collectively, this tells us that significant changes in prescription statistics were noted for 7 months after the pandemic's onset as compared to the 15 months period before.

Subgroup analysis (Supplemental Results Table S4, Table S5) shows that there is a growing preference for the 6‐month formulation (37% increase for Decapeptyl SR 22.5 mg inj vials and 20% increase for Triptorelin embonate 22.5 mg inj vials) as the pandemic progresses, with a related decreased preference for the 1‐month products. This indicates switching within the drug category in preference for agents with longer posology. The 6‐month formulations are normally reserved for female patients, but our analysis suggests that practice is adapting pragmatically.

No seasonal effects are expected because follow‐up appointments should be anticipated and prescheduled. In light of this, unexpectedly low prescription volumes were identified in August 2020 (goserelin 17 661, leuprorelin 15 987, and triptorelin acetate 6375).

### 5.3 By price of medicines

The actual cost of medicines shows minimal variation in “Results Supplemental Table S3” that tracks the total quantity changes described above. This suggests that prices of individual medicines have remained stable over the study period.

### 5.4 By location

The EDP presents data by “regional office name.” Nomenclature for regional territories except London was modified in April 2020, making it difficult to make direct comparisons across regions before and after this period. However sufficient clarity is provided to permit the re‐aggregation of the data (April‐July 20) to allow for direct comparison with prior periods (North West + North East and Yorkshire = North of England, Midlands = Midlands and East of England, South East + South West = South of England and London), see “Results Supplemental Figure S1 and Table S4.” This shows variation was substantial in some regions, though not statistically significant, with some unlabeled records. In all cases, the October 2020 statistics are lower “year on year” and as compared to January 2019 across regions.

Changes in population structure do not confound findings: In 2019, there were 712 680 live births in the UK (731 213 in 2018) and 604 707 deaths (616 014 in 2018),^30^ net growth (107 973). Provisional statistics put 608 016 deaths in England and Wales in 2020.^31^ The number of births for the first three quarters in England and Wales for 2020 were 464 437 (481 767 in 2019).^32^ Extrapolating provisional estimates (Birth 580 546, Death 760 020 Net −179 474) gives a net decline of approximately 180 000. Hence, the assumption that the cohort was “constant” remains valid.

## 6 DISCUSSION

Our analysis reflects approximately 47 000 prescriptions per month: over the study period, this probably reflects all PCa patients and other patient groups. We present data from the full first UK wave (first lockdown March 23 to October 31, 2020) where travel restrictions mean that this analysis presented a near complete first wave analysis. Findings are concerning and tell us that a significant number of patients may not have used their PCa medicines as expected. Switching from one medicine to another is unusual for these categories of medicines, unless there is inadequate testosterone suppression which is uncommon. Stopping gonadorelins is not evidenced by starting other agents in this category in the dataset. As a result, the gap we identify is likely to be an “unmet” need. Lockdowns may have a negative effect on primary healthcare from our data, because the “stay‐at‐home” message is translating into lower rates of adherence. We suspect that future lockdowns (regional or national) translate into healthcare avoidance by patients.

The GnRHa use in PCa is palliative. This combined with the fact that testosterone levels may remain suppressed for some time after GnRHa cessation make significant morbidity to PCa patients unlikely—at least in the short term. Low testosterone concentrations can be maintained for some time after GnRH cessation. However, there is some evidence that allowing testosterone levels to exceed 1.1 nmol/L predicts a lower survival, free of androgen independent progression.^33^ Stopping androgen depletion therapy (ADT) may not necessarily lead to raised levels of testosterone with worse outcomes and that in appropriate circumstances intermittent ADT may be a valid treatment strategy. However, as shown by Morote et al^33^ and more recently by Saad et al^34^ testosterone breakthroughs during ADT likely result in worse clinical outcomes and should be avoided.

Intermittent ADT could provide a flexible strategy and gives a valuable opportunity to plan for subsequent waves of the pandemic and for the longer‐term management of the clinical care of patients whose disease may have progressed further, than would have been otherwise anticipated. As UK public vaccination began in January 2021, it will be interesting to study the impact on adherence in future dated data. As postvaccination linked social restrictions are eased, we would rationally expect patients to return to their doctors for reassessment. However, there is a small risk that these patients may structurally avoid healthcare either because the “stay‐at‐home messaging” is too effective, they experience emotional guilt from having survived the pandemic, and other patient narratives or live experiences that are understudied. These are complex systems that cannot be easily modeled.

We know, in gynecology, that GnRH analogues are used in endometriosis and in vitro fertilization (IVF). For endometriosis, the duration of use is usually 6 months within secondary care with some GP issued repeat prescriptions. All IVF management is either in tertiary care or private settings. Therefore, the changes in the pattern of prescription for GnRH analogues observed in this study pertains predominantly to PCa patients, which is a cause for concern. The female patient population is a small proportion and has been stable historically. So changes in this patient group are unlikely to explain the variations we observe. There is no evidence to suggest that prescriptions in women who be handled any differently to those in men.

Prescription volumes have declined and even if this is not linked to significant morbidity now, it is a proxy for medicines‐supply or patient‐access and an important avenue for further enquiry. For the first time, we present data on prescription and regional variations during this pandemic for medicines licensed for the treatment of PCa. This provides an early signal for potentially deteriorating medium to longer term health in this group of patients. The data provide immediate decision‐making capacity which can be implemented to mitigate clinical risks for these patient groups.

Anecdotally, this could be for a variety of reasons including higher mortality within this patient group from COVID‐19, patient reluctance to leave home especially if they are shielding, and if they are on “patient triggered follow up” regimes with an emphasis put on the patent to contact the GP surgery for the next injection rather than vice versa. GP factors may also play a role and although surgeries normally have processes in place to enable patient recall and offer treatment at home where appropriate. However, in a pandemic, these processes may be less robust and patient perception has been that contacting their GP surgeries is very difficult and even that they are “closed.”

While we cannot be certain, the results suggest the possibility of a causal relation between the pandemic‐related healthcare changes and changes to prescription volumes. Our analysis cannot rule out other possible causal explanatory factors.

As researchers, we encourage improvement in the documentation and data‐structure of the dataset used. The need for error‐free data, its completeness, and the importance of documenting indications for medications is vital in facilitating better research that allows granular targeting of patient groups, as we have done here. Data collection, duration, and completeness requires that the data be representative of practice across the UK and should incorporate datasets from Scotland, Wales, and Northern Ireland income parable, or interoperable data presentations for completeness. This would allow early detection of regional variations to care.

There has also been significant disruption to the supply chain before and during COVID, coupled with pharmacy reimbursement‐renegotiations and manufacturing issues affecting medicine supply across Europe, but which do not affect these medicines.^35^, ^36^, ^37^ “Per oral” formulations may become available in the near future, where compliance rates may increase as a result of decoupling the use of injectable GnRHa from clinical settings. Year‐long depot‐injections should also be considered for development by manufacturers. This has the potential to substantially improve compliance rates since patients do not have healthcare contact outside of collecting medicines at a pharmacy/single annual visit. Similarly, investments in educating and training of patients to self‐administer therapy may be important, as with insulin‐dependent diabetics.

While the pandemic has provided an opportunity for digital consultations and remote supervision, they have come with added uncertainty and anxiety for patients. Changes to routine have the potential for negative consequences. Digital consultations have the potential to create digital‐barriers to care. This may be especially problematic for elderly patients. Adherence concerns and access to timely prescription refills may occur for a variety of reasons detailed above. Telephone triage may have substituted for the standard practice of a physical examination, PSA blood‐tests or annual review. Of key concern are new patients who have either delayed diagnoses or have been newly initiated on these medications and have failed to return as a consequence of the pandemic.

Another consideration is the “prescribing” vs. “dispensing” practice: we known that varied prescribing practice occurred, deviating from routine issuance of a “28‐day” prescription (in some cases, people were issued up to 6 months’ worth of medication). From our data, it is clear that this is modest. We know that the medicine's supply‐chain can only fulfil an excess demand by 2 weeks national average. In pharmacy practice, this often means that prescriptions are partly fulfilled with an “owing” or balance outstanding to the patient, which is settled when stocks become available. However, since pharmacies are contractors to the NHS, they are highly reliant on monthly reimbursements. Hence, these types of prescriptions (eg 6 months) are immediately sent for reimbursement and so will appear in national prescription‐statistics. In reality, patients may not have the medicines that appear to be fully‐dispensed to them, and the unmet medical need that we describe may be more widespread than first appears. Collectively, there may be instances across the country where patients have sub‐optimal disease control, where underlying complications may escalate.

While this analysis provides important insight, it can only be descriptive and further work is needed to explore the underlying reasons for the trends observed and the implications for patients. The numbers we present are a fraction of the directly attributable costs of PCa management. They do not cover the costs of complications, surgery, and onward care including the health‐burden borne by family or carers.

## 7 IMPLICATIONS FOR PUBLIC HEALTH

While we find statistically significant data, this may not be clinically relevant immediately for all affected patients. However, these data trends may continue in the second and third wave of lockdowns in the UK and can become entrenched, which would mean that a “clinical urgency” may appear unexpectedly.

This has implications for clinical practice—we encourage prescribers to maintain clear documentation of offered follow‐up and alternative care provided to guard against negligence cases and to actively think about patient‐lists. Gynecological and urological specialists have an important leadership role to guide policy and the direction of patient care within a multidisciplinary team in concert primary care which is rapidly reconfiguring. The evidence we present may support them in considering alternative models of care for their patients, including exploring innovative practices. Bilateral orchidectomy remains an option for locally advanced and metastatic cancers, but is unlikely to apply to the majority population. It is likely that delayed diagnosis and deprioritization of patient lists may mean that patient numbers (and therefore prescriptions) will substantially rise in the future. Population demographics also support increased GnRHa use in an aging cohort and workload and workforce preparedness should be considered.

## 8 STRENGTHS

There are several strengths to this study. For the first time, we report the impact on prescription volumes of medicines licensed for PCa in England during a global pandemic. Strengths of this study include being evidence‐based using real world data. One of the strengths of ITS studies is that they are generally unaffected by typical confounding variables which remain fairly constant, such as population age distribution or socioeconomic status, as these only change relatively slowly over time. Nevertheless, ITS can be affected by time‐varying confounders (eg, excess mortality) that change more rapidly.^38^

## 9 LIMITATIONS

We acknowledge that the indications for the medicines analyzed are unknown and no clear correlation with adverse outcomes appears in the literature.

Limitations pertain to the timeframe, completeness, and quality of the data. We have extracted government data however, they have not been independently verified as complete, accurate, and are subject to revision. The analysis is descriptive with no adjustments, for changes in population structure (age, disease prevalence, social deprivation scores) which could impact prescriptions between periods and within regions. Hospital statistics are not represented in our analysis. Confirmed diagnoses or prescription indications as well as linked data were unavailable to us. The linked data (eg, demographics and hospital admissions) with demographics (eg, by sex, age category) are unavailable freely, so it is difficult to quantify the proportion of GnRH scripts that are for PCa vs other conditions.

## 10 FUTURE STUDIES

This study generates an early warning signal from real‐world data on patients’ lives and provides a model for future pandemic preparedness. Future studies must consider the impact on patients’ lives with respect to disease progression, including over the life course of this pandemic. It is important to consider subsequent periods and interval between lockdowns to fully assess the potential impact to PCa patients. Future studies should examine whether routine PSA blood tests were conducted and if not, then what does that structurally missing data imply. Health economic analyses should be conducted.

## 11 CONCLUSION

There has been a decline in PCa prescription medicines dispensed and this may have occurred for a variety of reasons that we do not fully understand. We do know that not using these medicines has the potential to result in increased morbidity and mortality. Extra effort may be needed to help these patients.

## CONFLICT OF INTERESTS

All authors have completed the ICMJE uniform disclosure form at www.icmje.org/coi_disclosure.pdf and declare: no financial relationships or activities that could appear to have influenced the submitted work.

## AUTHOR CONTRIBUTIONS

RB (corresponding author) conducted the literature search, study conception and design data analysis, statistical analysis and interpretation of data, manuscript preparation, editing and revision and submitted the final version of the paper. RB provided technical expertise with data extraction, cleaning, manipulation and data for final analysis. KD and BB considered the clinical impact and consequences of our findings on this patient population.

## Supporting information

Supplementary Material

Supplementary Material

Supplementary Material

Supplementary Material

Supplementary Material

Supplementary Material

Video S1
