# Supplementary material for: Gonadorelins adherence in prostate cancer: A time‐series analysis of England’s national prescriptions during the COVID‐19 pandemic (from Jan 2019 to Oct 2020)
Source: BJUI Compass. 2021 Aug 19;2(6):419–27. doi: 10.1002/bco2.101 (PMC8427122; doi:10.1002/bco2.101)
Supplement: Supplementary file 1 — Supplementary Material [file BCO2-2-419-s001.pdf]

```
TSMODEL
  /MODELSUMMARY  PRINT=[MODELFIT]
  /MODELSTATISTICS  DISPLAY=YES MODELFIT=[ SRSQUARE]
  /MODELDETAILS  PRINT=[ PARAMETERS]
  /SERIESPLOT OBSERVED FORECAST
  /OUTPUTFILTER DISPLAY=ALLMODELS
  /SAVE  PREDICTED(Predicted) LCL(LCL) UCL(UCL)
  /AUXILIARY  CILEVEL=95 MAXACFLAGS=24
  /MISSING USERMISSING=EXCLUDE
  /MODEL DEPENDENT=Degarelix PreDegarelix PostDegarelix PreDegarelix_A PostDegarelix_A
   Goserelinacetate PreGoserelinacetate PostGoserelinacetate PreGoserelinacetate_A
   PostGoserelinacetate_A Leuprorelinacetate PreLeuprorelinacetate PostLeuprorelinacetate
   PreLeuprorelinacetate_A PostLeuprorelinacetate_A TriptorelinAcetate PreTriptorelinAcetate
   PostTriptorelinAcetate PreTriptorelinAcetate_A PostTriptorelinAcetate_A Triptorelinembonate
   PreTriptorelinembonate PostTriptorelinembonate PreTriptorelinembonate_A PostTriptorelinembona

   TotalQuantity_A PreTotalQuantity PostTotalQuantity PreTotalQuantity_A PostTotalQuantity_A
   ActualCost_A PreActualCost PostActualCost PreActualCost_A INDEPENDENT=Phase SeriesTime
riod
   Interact
     PREFIX='Model'
  /ARIMA AR=[1]  DIFF=0  MA=[0]  ARSEASONAL=[0]  DIFFSEASONAL=0  MASEASONAL=[0]
     TRANSFORM=NONE  CONSTANT=YES
  /TRANSFERFUNCTION VARIABLES=SeriesTimePeriod  NUM=[0] DENOM=[0] DIFF=0 NUMSEAS
AL=[0]
   DENOMSEASONAL=[0] DIFFSEASONAL=0 DELAY=0   TRANSFORM=NONE
  /TRANSFERFUNCTION VARIABLES=Phase  NUM=[0] DENOM=[0] DIFF=0 NUMSEASONAL=[0] D
NOMSEASONAL=[0]
   DIFFSEASONAL=0 DELAY=0   TRANSFORM=NONE
  /TRANSFERFUNCTION VARIABLES=Interact  NUM=[0] DENOM=[0] DIFF=0 NUMSEASONAL=[0]
NOMSEASONAL=[0]
   DIFFSEASONAL=0 DELAY=0   TRANSFORM=NONE
  /AUTOOUTLIER DETECT=OFF.
```
