# Supplementary material for: Gonadorelins adherence in prostate cancer: A time‐series analysis of England’s national prescriptions during the COVID‐19 pandemic (from Jan 2019 to Oct 2020)
Source: BJUI Compass. 2021 Aug 19;2(6):419–27. doi: 10.1002/bco2.101 (PMC8427122; doi:10.1002/bco2.101)
Supplement: Supplementary file 2 — Supplementary Material [file BCO2-2-419-s005.docx]

Supplemental Results (Table 1, Table 2, Figure 1, Table 3)

Table 1 Total Quantity in absolute numbers, unless indicated.

| Month | Goserelin acetate | Leuprorelin acetate | Triptorelin (Acetate) | Triptorelin embonate | Monthly Subtotal |
| --- | --- | --- | --- | --- | --- |
| Constituents | Goserelin 3.6mg & 10.8mg implant pre-filled syringes; Zoladex 3.6mg implant SafeSystem pre-filled syring & Zoladex LA 10.8mg implant SafeSystem pre-filled sy | Leuprorelin 3.75mg 10.72mg, 11.25mg, 22.5mg implant pre-filled syringes & vials, Lutrate 1 month Depot 3.75mg & 3 month Depot 22.5mg inj vials,  Prostap SR 3.75mg inj vials & SR DCS 3.75mg inj pre-filled syringes,  Prostap 3 DCS 11.25mg inj pre-filled syringes & Depot 11.25mg inj vials | Decapeptyl SR 3mg, 11.25mg inj vials, Gonapeptyl Depot 3.75mg inj pre-filled syringes Triptorelin 3mg, 11.25mg inj vials, Triptorelin acetate 3.75mg inj pre-filled syringes | Decapeptyl SR 22.5mg inj vials, Triptorelin embonate 22.5mg inj vials, Salvacyl 11.25mg inj vials |  |
| Jan-19 | 20630 | 18583 | 7316 | 899 | 49088 |
| Feb-19 | 18178 | 16614 | 6356 | 856 | 43433 |
| Mar-19 | 19830 | 17828 | 6961 | 887 | 47092 |
| Apr-19 | 19478 | 17584 | 6886 | 881 | 46434 |
| May-19 | 20493 | 18599 | 7216 | 909 | 48922 |
| Jun-19 | 18789 | 17314 | 6633 | 856 | 45165 |
| Jul-19 | 20660 | 18238 | 7320 | 898 | 48730 |
| Aug-19 | 19830 | 18100 | 7019 | 876 | 47557 |
| Sep-19 | 19492 | 17459 | 6815 | 906 | 46415 |
| Oct-19 | 20763 | 18884 | 7333 | 989 | 49839 |
| Nov-19 | 19648 | 17387 | 6982 | 889 | 46680 |
| Dec-19 | 19862 | 17934 | 6974 | 977 | 47535 |
| Jan-20 | 21358 | 19617 | 7832 | 938 | 51692 |
| Feb-20 | 18982 | 17029 | 6775 | 885 | 45486 |
| Mar-20 | 20269 | 17829 | 7179 | 949 | 48096 |
| Apr-20 | 20874 | 19268 | 7335 | 1202 | 50712 |
| May-20 | 19149 | 17315 | 6866 | 1079 | 46195 |
| Jun-20 | 19646 | 17921 | 7009 | 1129 | 47584 |
| Jul-20 | 20003 | 18122 | 7118 | 1049 | 48249 |
| Aug-20 | 17661 | 15987 | 6375 | 939 | 42732 |
| Sep-20 | 19356 | 17133 | 6819 | 1094 | 46370 |
| Oct-20 | 19497 | 17670 | 7225 | 1208 | 47632 |

Table 2 Actual Cost in £millions (Pound Sterling/Great British Pound).

| CHEMICAL_SUBSTANCE | Goserelin acetate | Leuprorelin acetate | Triptorelin (Acetate) | Triptorelin embonate | Monthly Subtotal |
| --- | --- | --- | --- | --- | --- |
| Jan-19 | 3.1 | 3.3 | 1.2 | 0.3 | 8.2 |
| Feb-19 | 2.8 | 2.9 | 1.0 | 0.3 | 7.3 |
| Mar-19 | 3.0 | 3.2 | 1.1 | 0.3 | 7.8 |
| Apr-19 | 3.0 | 3.1 | 1.1 | 0.3 | 7.8 |
| May-19 | 3.1 | 3.3 | 1.2 | 0.3 | 8.1 |
| Jun-19 | 2.9 | 3.1 | 1.1 | 0.3 | 7.5 |
| Jul-19 | 3.2 | 3.2 | 1.2 | 0.3 | 8.1 |
| Aug-19 | 3.0 | 3.2 | 1.1 | 0.3 | 7.9 |
| Sep-19 | 3.0 | 3.1 | 1.1 | 0.3 | 7.7 |
| Oct-19 | 3.1 | 3.4 | 1.2 | 0.4 | 8.3 |
| Nov-19 | 2.9 | 3.1 | 1.1 | 0.3 | 7.7 |
| Dec-19 | 3.0 | 3.2 | 1.1 | 0.4 | 7.9 |
| Jan-20 | 3.2 | 3.5 | 1.3 | 0.4 | 8.6 |
| Feb-20 | 2.8 | 3.0 | 1.1 | 0.3 | 7.5 |
| Mar-20 | 3.0 | 3.2 | 1.2 | 0.4 | 8.0 |
| Apr-20 | 3.2 | 3.5 | 1.2 | 0.5 | 8.6 |
| May-20 | 2.9 | 3.1 | 1.1 | 0.4 | 7.8 |
| Jun-20 | 3.0 | 3.3 | 1.2 | 0.4 | 8.1 |
| Jul-20 | 3.0 | 3.3 | 1.2 | 0.4 | 8.1 |
| Aug-20 | 2.7 | 2.9 | 1.1 | 0.4 | 7.2 |
| Sep-20 | 2.9 | 3.1 | 1.1 | 0.4 | 7.8 |
| Oct-20 | 2.9 | 3.2 | 1.2 | 0.5 | 8.0 |

Figure 1 Regional Total Quantity by region

Table 3 Total Quantity by region

| Region | North West, North East and Yorkshire, | Midlands, East of England, | South East, South West | London | Unidentified | Sub-total |
| --- | --- | --- | --- | --- | --- | --- |
| Jan-19 | 12803 | 16652 | 14601 | 5031 | 1 | 49088 |
| Feb-19 | 11354 | 14570 | 13136 | 4371 | 2 | 43433 |
| Mar-19 | 12474 | 15630 | 14202 | 4783 | 3 | 47092 |
| Apr-19 | 12264 | 15673 | 13767 | 4726 | 4 | 46434 |
| May-19 | 13264 | 16429 | 14526 | 4702 | 1 | 48922 |
| Jun-19 | 12047 | 15026 | 13637 | 4454 | 1 | 45165 |
| Jul-19 | 12907 | 16557 | 14148 | 5115 | 3 | 48730 |
| Aug-19 | 12786 | 15909 | 14064 | 4797 | 1 | 47557 |
| Sep-19 | 12308 | 15496 | 13915 | 4689 | 7 | 46415 |
| Oct-19 | 13345 | 16789 | 14712 | 4991 | 2 | 49839 |
| Nov-19 | 12721 | 15469 | 13809 | 4677 | 4 | 46680 |
| Dec-19 | 12496 | 16225 | 14068 | 4745 | 1 | 47535 |
| Jan-20 | 13718 | 17271 | 15511 | 5188 | 4 | 51692 |
| Feb-20 | 11949 | 15328 | 13493 | 4711 | 5 | 45486 |
| Mar-20 | 13013 | 15988 | 13973 | 5121 | 1 | 48096 |
| Apr-20 | 13567 | 17062 | 15139 | 4940 | 4 | 50712 |
| May-20 | 11992 | 15805 | 13829 | 4568 | 1 | 46195 |
| Jun-20 | 12796 | 16003 | 14020 | 4762 | 3 | 47584 |
| Jul-20 | 12758 | 16346 | 14478 | 4664 | 3 | 48249 |
| Aug-20 | 11743 | 14247 | 12556 | 4175 | 11 | 42732 |
| Sep-20 | 12719 | 15242 | 13638 | 4767 | 4 | 46370 |
| Oct-20 | 12560 | 16106 | 14130 | 4828 | 8 | 47632 |
