# Supplementary material for: Gonadorelins adherence in prostate cancer: A time‐series analysis of England’s national prescriptions during the COVID‐19 pandemic (from Jan 2019 to Oct 2020)
Source: BJUI Compass. 2021 Aug 19;2(6):419–27. doi: 10.1002/bco2.101 (PMC8427122; doi:10.1002/bco2.101)
Supplement: Supplementary file 3 — Supplementary Material [file BCO2-2-419-s004.pdf]

Supplemental Results (Table 4, Table 5).

Posology	BNF_DESCRIPTION	BNF_CODE	Jan-19	Feb-19	Mar-19	Apr-19	May-19	Jun-19	Jul-19	Aug-19	Sep-19	Oct-19	Nov-19	Dec-19	Jan-20	Feb-20	Mar-20	Apr-20	May-20	Jun-20	Jul-20	Aug-20	Sep-20	Oct-20	Change (%) Oct20/Jan19	Trend
Three month	Prostap 3 DCS 11.25mg inj pre-filled syringes	0803042N0BBADAD	9905	8722	9540	9451	9949	9416	9791	9712	9543	10303	9369	9741	10656	9088	9703	10855	9883	10323	10441	9202	9829	10159	3%	
Three month	Zoladex LA 10.8mg implant SafeSystem pre-filled sy	0803042K0BBABAB	7456	6469	7038	7119	7409	6701	7469	7063	7027	7301	6906	7074	7493	6671	7098	7643	6923	7209	7317	6388	7076	6978	-6%	
One month	Zoladex 3.6mg implant SafeSystem pre-filled syring	0803042K0BBAAAA	5448	4758	5260	5161	5480	5045	5652	5526	5394	5749	5586	5601	6149	5554	5768	5649	5493	5532	5758	5115	5576	5627	3%	
Three month	Decapeptyl SR 11.25mg inj vials	0803042P0BBABAC	4882	4260	4635	4653	4882	4529	4926	4774	4608	5035	4780	4701	5410	4571	4979	5131	4830	5040	5042	4546	4854	5147	5%	
Three month	Leuprorelin 11.25mg inj pre-filled syringes	0803042N0AAADAD	4336	3907	4095	3993	4250	3941	4232	4313	3898	4274	4036	4006	4478	3781	4160	4476	3944	4135	4185	3615	3952	4061	-6%	
Three month	Goserelin 10.8mg implant pre-filled syringes	0803042K0AAABAB	4546	4087	4323	4186	4357	4057	4315	4097	4083	4374	3994	3970	4330	3734	4039	4306	3833	3883	3933	3435	3806	3778	-17%	
One month	Goserelin 3.6mg implant pre-filled syringes	0803042K0AAAAAA	3180	2864	3209	3012	3247	2986	3224	3144	2988	3339	3162	3217	3386	3023	3364	3276	2900	3022	2995	2723	2898	3114	-2%	
One month	Prostap SR DCS 3.75mg inj pre-filled syringes	0803042N0BBACAC	2781	2569	2759	2743	2854	2584	2789	2683	2723	2827	2581	2756	2970	2763	2583	2652	2329	2306	2355	2142	2308	2348	-16%	
One month	Decapeptyl SR 3mg inj vials	0803042P0BBAAAA	1261	1076	1193	1173	1230	1092	1296	1190	1235	1254	1241	1256	1403	1244	1221	1182	1105	1073	1141	1014	1050	1162	-8%	
One month	Leuprorelin 3.75mg inj pre-filled syringes	0803042N0AAACAC	1424	1308	1359	1346	1494	1329	1385	1364	1264	1446	1391	1428	1502	1389	1376	1277	1157	1150	1137	1024	1039	1100	-23%	
Six months	Decapeptyl SR 22.5mg inj vials	0803042S0BCAAAB	771	701	719	734	756	731	764	730	784	814	741	812	797	760	788	1033	911	961	913	810	917	1055	37%	
Three month	Triptorelin 11.25mg inj vials	0803042P0AAACAC	591	519	596	537	557	527	549	556	520	545	520	529	537	510	541	528	503	484	506	450	515	507	-14%	
One month	Gonapeptyl Depot 3.75mg inj pre-filled syringes	0803042P0BCAAAB	367	297	310	315	347	298	322	303	274	269	258	268	280	269	248	300	243	247	234	218	228	246	-33%	
Six months	Triptorelin embonate 22.5mg inj vials	0803042S0AAABAB	126	152	163	146	150	121	133	143	121	172	148	163	141	122	158	168	166	164	132	127	172	151	20%	
One month	Triptorelin acetate 3mg inj vials	0803042P0AAAAAA	123	128	143	130	116	112	138	137	117	163	119	143	124	115	122	116	126	104	130	107	108	123	0%	
One month	Triptorelin acetate 3.75mg inj pre-filled syringes	0803042P0AAABAB	92	76	84	78	84	75	89	59	61	67	64	77	78	66	68	78	59	61	65	40	64	40	-57%	
Three month	Salvacyl 11.25mg inj vials	0803042S0BDAAAC	2	3	5	1	3	4	1	3	1	3	0	2	0	3	3	1	2	4	4	2	5	2	0%	
One month	Leuprorelin 3.75mg inj vials	0803042N0AAAAAA	66	45	53	42	43	36	35	25	30	28	7	1	6	6	4	1	1	1	1	2	3	1	-98%	
Three month	Lutrate 3 month Depot 22.5mg inj vials	0803042N0BCABAE	46	47	13	8	3	2	1	0	0	1	1	0	0	0	0	0	0	0	1	0	0	1	-98%	
Three month	Prostap 3 Depot 11.25mg inj vials	0803042N0BBABAB	1	2	0	0	0	0	1	0	0	0	0	0	1	0	0	3	0	1	1	0	0	0	-100%	
Three month	Leuprorelin 11.25mg inj vials	0803042N0AAABAB	1	0	4	1	2	1	1	0	0	1	1	2	2	1	1	1	1	4	0	0	1	0	-100%	
One month	Lutrate 1 month Depot 3.75mg inj vials	0803042N0BCAAAA	3	4	1	0	1	3	1	1	0	2	1	0	1	0	2	0	0	1	0	2	1	0	-100%	
Three month	Leuprorelin 22.5mg inj vials	0803042N0AAAEAE	20	10	4	0	3	1	2	2	1	2	0	0	0	1	0	2	0	0	0	0	0	0	-100%	
One month	Prostap SR 3.75mg inj vials	0803042N0BBAAAA	0	0	0	0	0	1	0	0	0	0	0	0	1	0	0	0	0	0	1	0	0	0		
Three month	Leuprorelin 10.72mg implant pre-filled syringes	0803042N0AAAFAF	0	0	0	0	0	0	0	0	0	0	0	0	0	0	0	1	0	0	0	0	0	0		
	Supplemental Results Table 4	Total Quantity	49088	43433	47092	46434	48922	45165	48730	47557	46415	49839	46680	47535	51692	45486	48096	50712	46195	47584	48249	42732	46370	47632	97%	

BNF_DESCRIPTION	BNF_CODE	Jan-19	Feb-19	Mar-19	Apr-19	May-19	Jun-19	Jul-19	Aug-19	Sep-19	Oct-19	Nov-19	Dec-19	Jan-20	Feb-20	Mar-20	Apr-20	May-20	Jun-20	Jul-20	Aug-20	Sep-20	Oct-20		Trend
Prostap 3 DCS 11.25mg inj pre-filled syringes	0803042N0BBADAD	2.08	1.83	2.00	1.98	2.09	1.98	2.05	2.04	2.00	2.16	1.97	2.04	2.24	1.91	2.03	2.28	2.07	2.17	2.19	1.93	2.06	2.13	103%	
Zoladex LA 10.8mg implant SafeSystem pre-filled sy	0803042K0BBABAB	1.63	1.41	1.54	1.55	1.62	1.46	1.63	1.54	1.53	1.59	1.51	1.55	1.64	1.46	1.55	1.67	1.51	1.57	1.60	1.40	1.54	1.52	94%	
Decapeptyl SR 11.25mg inj vials	0803042P0BBABAC	0.94	0.82	0.89	0.89	0.94	0.87	0.95	0.92	0.89	0.97	0.92	0.90	1.04	0.88	0.96	0.99	0.93	0.97	0.97	0.88	0.93	0.99	105%	
Leuprorelin 11.25mg inj pre-filled syringes	0803042N0AAADAD	0.91	0.82	0.86	0.84	0.89	0.83	0.89	0.90	0.82	0.90	0.85	0.84	0.94	0.79	0.87	0.94	0.83	0.87	0.88	0.76	0.83	0.85	94%	
Goserelin 10.8mg implant pre-filled syringes	0803042K0AAABAB	0.99	0.89	0.94	0.91	0.95	0.89	0.94	0.89	0.89	0.95	0.87	0.87	0.95	0.82	0.88	0.94	0.84	0.85	0.86	0.75	0.83	0.82	83%	
Decapeptyl SR 22.5mg inj vials	0803042S0BCAAAB	0.30	0.27	0.28	0.28	0.29	0.28	0.29	0.28	0.30	0.31	0.29	0.31	0.31	0.29	0.30	0.40	0.35	0.37	0.35	0.31	0.35	0.41	137%	
Zoladex 3.6mg implant SafeSystem pre-filled syring	0803042K0BBAAAA	0.33	0.31	0.34	0.34	0.36	0.33	0.37	0.36	0.35	0.37	0.36	0.36	0.40	0.36	0.38	0.37	0.36	0.36	0.37	0.33	0.36	0.37	111%	
Goserelin 3.6mg implant pre-filled syringes	0803042K0AAAAAA	0.19	0.19	0.21	0.20	0.21	0.19	0.21	0.20	0.19	0.22	0.21	0.21	0.22	0.20	0.22	0.21	0.19	0.20	0.19	0.18	0.19	0.20	105%	
Prostap SR DCS 3.75mg inj pre-filled syringes	0803042N0BBACAC	0.19	0.18	0.19	0.19	0.20	0.18	0.20	0.19	0.19	0.20	0.18	0.19	0.21	0.19	0.18	0.19	0.16	0.16	0.16	0.15	0.16	0.16	84%	
Triptorelin 11.25mg inj vials	0803042P0AAACAC	0.11	0.10	0.11	0.10	0.11	0.10	0.11	0.11	0.10	0.10	0.10	0.10	0.10	0.10	0.10	0.10	0.10	0.09	0.10	0.09	0.10	0.10	86%	
Leuprorelin 3.75mg inj pre-filled syringes	0803042N0AAACAC	0.10	0.09	0.10	0.09	0.10	0.09	0.10	0.10	0.09	0.10	0.10	0.10	0.11	0.10	0.10	0.09	0.08	0.08	0.08	0.07	0.07	0.08	77%	
Decapeptyl SR 3mg inj vials	0803042P0BBAAAA	0.08	0.07	0.08	0.08	0.08	0.07	0.08	0.08	0.08	0.08	0.08	0.08	0.09	0.08	0.08	0.08	0.07	0.07	0.07	0.07	0.07	0.07	92%	
Triptorelin embonate 22.5mg inj vials	0803042S0AAABAB	0.05	0.06	0.06	0.06	0.06	0.05	0.05	0.05	0.05	0.07	0.06	0.06	0.05	0.05	0.06	0.06	0.06	0.06	0.05	0.05	0.07	0.06	120%	
Gonapeptyl Depot 3.75mg inj pre-filled syringes	0803042P0BCAAAB	0.03	0.02	0.02	0.02	0.03	0.02	0.02	0.02	0.02	0.02	0.02	0.02	0.02	0.02	0.02	0.02	0.02	0.02	0.02	0.02	0.02	0.02	67%	
Triptorelin acetate 3mg inj vials	0803042P0AAAAAA	0.01	0.01	0.01	0.01	0.01	0.01	0.01	0.01	0.01	0.01	0.01	0.01	0.01	0.01	0.01	0.01	0.01	0.01	0.01	0.01	0.01	0.01	100%	
Triptorelin acetate 3.75mg inj pre-filled syringes	0803042P0AAABAB	0.01	0.01	0.01	0.01	0.01	0.01	0.01	0.00	0.00	0.01	0.00	0.01	0.01	0.01	0.01	0.01	0.00	0.00	0.00	0.00	0.00	0.00	43%	
Salvacyl 11.25mg inj vials	0803042S0BDAAAC	0.00	0.00	0.00	0.00	0.00	0.00	0.00	0.00	0.00	0.00	0.00	0.00	0.00	0.00	0.00	0.00	0.00	0.00	0.00	0.00	0.00	0.00	100%	
Lutrate 3 month Depot 22.5mg inj vials	0803042N0BCABAE	0.01	0.01	0.00	0.00	0.00	0.00	0.00	0.00	0.00	0.00	0.00	0.00	0.00	0.00	0.00	0.00	0.00	0.00	0.00	0.00	0.00	0.00	2%	
Leuprorelin 3.75mg inj vials	0803042N0AAAAAA	0.00	0.00	0.00	0.00	0.00	0.00	0.00	0.00	0.00	0.00	0.00	0.00	0.00	0.00	0.00	0.00	0.00	0.00	0.00	0.00	0.00	0.00	1%	
Prostap 3 Depot 11.25mg inj vials	0803042N0BBABAB	0.00	0.00	0.00	0.00	0.00	0.00	0.00	0.00	0.00	0.00	0.00	0.00	0.00	0.00	0.00	0.00	0.00	0.00	0.00	0.00	0.00	0.00	0%	
Leuprorelin 11.25mg inj vials	0803042N0AAABAB	0.00	0.00	0.00	0.00	0.00	0.00	0.00	0.00	0.00	0.00	0.00	0.00	0.00	0.00	0.00	0.00	0.00	0.00	0.00	0.00	0.00	0.00	0%	
Lutrate 1 month Depot 3.75mg inj vials	0803042N0BCAAAA	0.00	0.00	0.00	0.00	0.00	0.00	0.00	0.00	0.00	0.00	0.00	0.00	0.00	0.00	0.00	0.00	0.00	0.00	0.00	0.00	0.00	0.00	0%	
Leuprorelin 22.5mg inj vials	0803042N0AAAEAE	0.00	0.00	0.00	0.00	0.00	0.00	0.00	0.00	0.00	0.00	0.00	0.00	0.00	0.00	0.00	0.00	0.00	0.00	0.00	0.00	0.00	0.00	0%	
Prostap SR 3.75mg inj vials	0803042N0BBAAAA	0.00	0.00	0.00	0.00	0.00	0.00	0.00	0.00	0.00	0.00	0.00	0.00	0.00	0.00	0.00	0.00	0.00	0.00	0.00	0.00	0.00	0.00		
Leuprorelin 10.72mg implant pre-filled syringes	0803042N0AAAFAF	0.00	0.00	0.00	0.00	0.00	0.00	0.00	0.00	0.00	0.00	0.00	0.00	0.00	0.00	0.00	0.00	0.00	0.00	0.00	0.00	0.00	0.00		
Supplemental Results Table 5	Actual Cost in £millions	8.15	7.26	7.84	7.75	8.14	7.55	8.11	7.90	7.72	8.29	7.73	7.88	8.56	7.48	7.97	8.59	7.80	8.07	8.15	7.20	7.83	8.03	99%	
