# Supplementary material for: Gonadorelins adherence in prostate cancer: A time‐series analysis of England’s national prescriptions during the COVID‐19 pandemic (from Jan 2019 to Oct 2020)
Source: BJUI Compass. 2021 Aug 19;2(6):419–27. doi: 10.1002/bco2.101 (PMC8427122; doi:10.1002/bco2.101)
Supplement: Supplementary file 4 — Supplementary Material [file BCO2-2-419-s007.pdf]

Sensitivity Analysis: ARIMA Model Parameters

ARIMA(0,0,0)(0,0,0) - No Transformation	Column1	Parameter Est	SE	t	p-value
Goserelin acetate-Model_1	TimePeriod	42	53	0.795	0.437
Goserelin acetate-Model_1	Phase	3775	3214	1.174	0.256
Goserelin acetate-Model_1	Interact	-245	174	-1.407	0.176
Leuprorelin acetate-Model_2	TimePeriod	22	50	0.451	0.657
Leuprorelin acetate-Model_2	Phase	4690	3037	1.544	0.14
Leuprorelin acetate-Model_2	Interact	-276	165	-1.672	0.112
Triptorelin (Acetate)-Model_3	TimePeriod	23	21	1.121	0.277
Triptorelin (Acetate)-Model_3	Phase	826	1256	0.658	0.519
Triptorelin (Acetate)-Model_3	Interact	-61	68	-0.892	0.384
Triptorelin embonate-Model_4	TimePeriod	5	4	1.349	0.194
Triptorelin embonate-Model_4	Phase	329	223	1.479	0.156
Triptorelin embonate-Model_4	Interact	-10	12	-0.826	0.419

ARIMA(1,0,0)(0,0,0) - AR Lag 1, No Transformation	Column1	Parameter Est	SE	t	p-value
Goserelin acetate-Model_1	TimePeriod	59	29	2.037	0.058
Goserelin acetate-Model_1	Phase	4000	1909	2.095	0.051
Goserelin acetate-Model_1	Interact	-270	102	-2.643	**0.017**
Leuprorelin acetate-Model_2	TimePeriod	39	30	1.3	0.211
Leuprorelin acetate-Model_2	Phase	4735	1981	2.39	**0.029**
Leuprorelin acetate-Model_2	Interact	-292	106	-2.755	**0.014**
Triptorelin (Acetate)-Model_3	TimePeriod	30	12	2.552	**0.021**
Triptorelin (Acetate)-Model_3	Phase	1067	777	1.374	0.187
Triptorelin (Acetate)-Model_3	Interact	-79	42	-1.898	0.075
Triptorelin embonate-Model_4	TimePeriod	5	4	1.389	0.183
Triptorelin embonate-Model_4	Phase	335	244	1.37	0.189
Triptorelin embonate-Model_4	Interact	-10	13	-0.786	0.443

ARIMA(0,1,0)(0,0,0) -Difference 1, No Transformation	Column1	Parameter Est	SE	t	p-value
Goserelin acetate-Model_1	TimePeriod	37	103	0.362	0.722
Goserelin acetate-Model_1	Phase	-1538	5689	-0.27	0.79
Goserelin acetate-Model_1	Interact	56	311	0.18	0.859
Leuprorelin acetate-Model_2	TimePeriod	18	97	0.185	0.855
Leuprorelin acetate-Model_2	Phase	-326	5364	-0.061	0.952
Leuprorelin acetate-Model_2	Interact	9	293	0.03	0.976
Triptorelin (Acetate)-Model_3	TimePeriod	14	39	0.354	0.728
Triptorelin (Acetate)-Model_3	Phase	-1014	2140	-0.474	0.642
Triptorelin (Acetate)-Model_3	Interact	47	117	0.399	0.695
Triptorelin embonate-Model_4	TimePeriod	1	7	0.176	0.862
Triptorelin embonate-Model_4	Phase	58	366	0.157	0.877
Triptorelin embonate-Model_4	Interact	-2	20	-0.096	0.925

ARIMA(0,0,1)(0,0,0) - MA Lag 1, No Transformation	Column1	Parameter Est	SE	t	p-value**
Goserelin acetate-Model_1	TimePeriod	70	14	4.907	**0**
Goserelin acetate-Model_1	Phase	3846	1402	2.744	**0.014**
Goserelin acetate-Model_1	Interact	-274	73	-3.765	**0.002**
Leuprorelin acetate-Model_2	TimePeriod	31	14	2.23	**0.04**
Leuprorelin acetate-Model_2	Phase	4589	1656	2.772	**0.013**
Leuprorelin acetate-Model_2	Interact	-284	87	-3.272	**0.004**
Triptorelin (Acetate)-Model_3	TimePeriod	26	6	4.53	**0**
Triptorelin (Acetate)-Model_3	Phase	812	790	1.028	0.318
Triptorelin (Acetate)-Model_3	Interact	-64	40	-1.603	0.127
Triptorelin embonate-Model_4	TimePeriod	6	1	4.787	**0**
Triptorelin embonate-Model_4	Phase	522	200	2.615	**0.018**
Triptorelin embonate-Model_4	Interact	-22	11	-2.085	0.052

a Iteration did not converge during optimization. Model may be unreliable.

** p-values are <0.001; unless stated

ARIMA(2,0,0)(0,0,0) AR Lag 1, Lag 2, No Transformation	Column1	Parameter Est	SE	t	p-value**
Goserelin acetate-Model_1	TimePeriod	62	12	5.294	**0.000**
Goserelin acetate-Model_1	Phase	4703	864	5.442	**0.000**
Goserelin acetate-Model_1	Interact	-314	46	-6.823	**0.000**
Leuprorelin acetate-Model_2	TimePeriod	45	12	3.686	**0.002**
Leuprorelin acetate-Model_2	Phase	5167	887	5.826	**0.000**
Leuprorelin acetate-Model_2	Interact	-324	47	-6.855	**0.000**
Triptorelin (Acetate)-Model_3	TimePeriod	32	5	6.295	**0.000**
Triptorelin (Acetate)-Model_3	Phase	1329	374	3.554	**0.003**
Triptorelin (Acetate)-Model_3	Interact	-96	20	-4.811	**0.000**
Triptorelin embonate-Model_4	TimePeriod	6	2	2.816	**0.012**
Triptorelin embonate-Model_4	Phase	464	186	2.501	**0.024**
Triptorelin embonate-Model_4	Interact	-19	10	-1.868	0.080

** p-values are <0.001; unless stated

ARIMA(1,1,1)(0,0,0) AR Lag 1, Difference 1, MA Lag 1, No Transformation	Column1	Parameter Est	SE	t	p-value
Goserelin acetate-Model_1	TimePeriod	16	18	0.881	0.392
Goserelin acetate-Model_1	Phase	-1602	1578	-1.015	0.326
Goserelin acetate-Model_1	Interact	61	81	0.747	0.467
Leuprorelin acetate-Model_2	TimePeriod	18	20	0.884	0.391
Leuprorelin acetate-Model_2	Phase	-460	1715	-0.268	0.792
Leuprorelin acetate-Model_2	Interact	0	88	0.002	0.998
Triptorelin (Acetate)-Model_3	TimePeriod	13	9	1.5	0.154
Triptorelin (Acetate)-Model_3	Phase	-1062	784	-1.356	0.195
Triptorelin (Acetate)-Model_3	Interact	44	40	1.103	0.288
Triptorelin embonate-Model_4	TimePeriod	1	3	0.468	0.647
Triptorelin embonate-Model_4	Phase	47	341	0.137	0.893
Triptorelin embonate-Model_4	Interact	-3	17	-0.151	0.882

a Iteration did not converge during optimization. Model may be unreliable.

ARIMA(0,0,0)(1,0,0) AR Lag 1, Seasonal Lag 1, No Transformation	Column1	Parameter Est	SE	t	p-value
Goserelin acetate-Model_1	TimePeriod	48	42	1.146	0.268
Goserelin acetate-Model_1	Phase	4257	2916	1.46	0.163
Goserelin acetate-Model_1	Interact	-277	154	-1.792	0.091
Leuprorelin acetate-Model_2	TimePeriod	30	42	0.71	0.487
Leuprorelin acetate-Model_2	Phase	5110	2876	1.777	0.093
Leuprorelin acetate-Model_2	Interact	-305	153	-1.989	0.063
Triptorelin (Acetate)-Model_3	TimePeriod	28	15	1.958	0.067

Triptorelin (Acetate)-Model_3	Phase	911	1029	0.886	0.388
Triptorelin (Acetate)-Model_3	Interact	-69	54	-1.275	0.22
Triptorelin embonate-Model_4	TimePeriod	4	3	1.489	0.155
Triptorelin embonate-Model_4	Phase	339	235	1.443	0.167
Triptorelin embonate-Model_4	Interact	-10	12	-0.834	0.416

**ARIMA(0,0,0)(0,1,0) Seasonal Difference 1, No Transformation**	**Column1**	**Parameter Est**	**SE**	**t**	**p-value**
Goserelin acetate-Model_1	TimePeriod	-145	767	-0.188	0.857
Goserelin acetate-Model_1	Phase	2679	11453	0.234	0.823
Goserelin acetate-Model_1	Interact	-163	794	-0.205	0.845
Leuprorelin acetate-Model_2	TimePeriod	-517	742	-0.696	0.512
Leuprorelin acetate-Model_2	Phase	-1664	11074	-0.15	0.885
Leuprorelin acetate-Model_2	Interact	177	768	0.231	0.825
Triptorelin (Acetate)-Model_3	TimePeriod	-149	252	-0.591	0.576
Triptorelin (Acetate)-Model_3	Phase	-1193	3760	-0.317	0.762
Triptorelin (Acetate)-Model_3	Interact	78	261	0.3	0.774
Triptorelin embonate-Model_4	TimePeriod	12	54	0.214	0.837
Triptorelin embonate-Model_4	Phase	641	801	0.8	0.454
Triptorelin embonate-Model_4	Interact	-29	56	-0.515	0.625

**ARIMA(0,0,0)(0,0,1) MA, Seasonal Lag 1, No Transformation**	**Column1**	**Parameter Est**	**SE**	**t**	**p-value**
Goserelin acetate-Model_1	TimePeriod	48	42	1.146	0.268
Goserelin acetate-Model_1	Phase	4257	2916	1.46	0.162
Goserelin acetate-Model_1	Interact	-277	154	-1.792	0.091
Leuprorelin acetate-Model_2	TimePeriod	30	42	0.708	0.489
Leuprorelin acetate-Model_2	Phase	5102	2882	1.77	0.095
Leuprorelin acetate-Model_2	Interact	-304	154	-1.981	0.064
Triptorelin (Acetate)-Model_3	TimePeriod	28	16	1.714	0.105
Triptorelin (Acetate)-Model_3	Phase	917	1102	0.832	0.417
Triptorelin (Acetate)-Model_3	Interact	-69	58	-1.179	0.255
Triptorelin embonate-Model_4	TimePeriod	4	3	1.493	0.154
Triptorelin embonate-Model_4	Phase	372	230	1.614	0.125
Triptorelin embonate-Model_4	Interact	-12	12	-0.992	0.335

**ARIMA(1,0,0)(0,0,0) AR Lag 1, Natural Logarithm**	**Column1**	**Parameter Est**	**SE**	**t**	**p-value**	% change in y-medicine(Model) for a 1-unit increase in x-Column1		
Goserelin acetate-Model_1	TimePeriod	0.003	0.002	2.002	0.062	30%	i.e before Mar-20, Gos was growing 30% per month	
Goserelin acetate-Model_1	Phase	0.205	0.099	2.075	0.054	2275%	at Mar-20, it jumped to 2275%,	
Goserelin acetate-Model_1	Interact	-0.014	0.005	-2.615	0.018	-139%	then, in the period after, Gos was growing at -139% per month	This is stat sig.
Leuprorelin acetate-Model_2	TimePeriod	0.002	0.002	1.256	0.226	20%	i.e before Mar-20, LA was growing 20% per month	
Leuprorelin acetate-Model_2	Phase	0.266	0.112	2.376	0.03	3047%	at Mar-20, it jumped to 3047%,	This is stat sig.
Leuprorelin acetate-Model_2	Interact	-0.016	0.006	-2.737	0.014	-159%	then, in the period after, La was growing at -159% per month	This is stat sig.
Triptorelin (Acetate)-Model_3	TimePeriod	0.004	0.002	2.54	0.021	40%	i.e before Mar-20, TA was growing 40% per month	This is stat sig.
Triptorelin (Acetate)-Model_3	Phase	0.156	0.111	1.406	0.178	1688%	at Mar-20, it jumped to 1688%,	
Triptorelin (Acetate)-Model_3	Interact	-0.011	0.006	-1.924	0.071	-109%	then, in the period after, Gos was growing at -109% per month	
Triptorelin embonate-Model_4	TimePeriod	0.006	0.003	1.632	0.121	60%		
Triptorelin embonate-Model_4	Phase	0.340	0.228	1.494	0.153	4049%		
Triptorelin embonate-Model_4	Interact	-0.011	0.012	-0.906	0.378	-109%		

Coefficient transformation into ?% change

Ref: Benoit K. Linear regression models with logarithmic transformations. London School of Economics, London. 2011 Mar 17;22(1):23-36.

(March - 0, April - 1)
Time, phase and interact are all included in the regression.

the coefficient for 'time' gives us the slope of the regression line pre-intervention — BEFORE
the coefficient for 'phase' gives us the change in intercept — Step-change
the coefficient for 'interact' gives us the change in slope pre and post intervention — AFTER

This is explained below:

If the coefficient for time is β_1, for phase is β_2 and for interact is β_3 then the regression model is:

Outcome= constant + $\beta_1$time + $\beta_2$phase + $\beta_3$interact

Therefore, **pre** intervention becomes:

Outcome = constant + $\beta_1$time

And **post** intervention becomes:

Outcome= constant + $\beta_1$time + β_2 + $\beta_3$interact = (constant + β_2) + (β_1 + β_3) time (as time and interact are the same post intervention)

Therefore, the difference in constant (intercept) pre and post intervention is β_2 and the difference in slope is β_3.
