# Supplementary material for: Gonadorelins adherence in prostate cancer: A time‐series analysis of England’s national prescriptions during the COVID‐19 pandemic (from Jan 2019 to Oct 2020)
Source: BJUI Compass. 2021 Aug 19;2(6):419–27. doi: 10.1002/bco2.101 (PMC8427122; doi:10.1002/bco2.101)
Supplement: Supplementary file 6 — Supplementary Material [file BCO2-2-419-s006.docx]

Supplemental Methods

## Data Import & Cleaning

The EPD data from Feb 2019 to July 2020 consists of 333,459,762 rows of data. This is an unworkable number of datapoints for most analysis packages and workstations due to sheer size and software limitations.

Filtering the 333 million+ rows of data is achieved with the use of a relational database join. A list of BNF codes was created and stored within a table. The ‘reqDrugsForReporting’ table contained xxx BNF codes. We created a relationship between the two tables using an ‘inner join’. This effectively filters the EPD table when a ‘view’ is built upon this relationship. With the database join in place, a view of the data results in a subset of the EPD data which is 2,555,396 rows.

This subset result is further aggregated by acquiring a sum of ‘Total Quantity’, ‘Actual Cost’ and ‘Number of items’. This is broken down by: ‘BNF Code’, by ‘Month’, ‘Regional Office Name’ and ‘STP Name’ (See Figure 1).

Though other fields are present within the dataset they do not constitute aggregation dimensions but are included for sorting and analysis. The final export results in 24,381 rows of data for analysis.


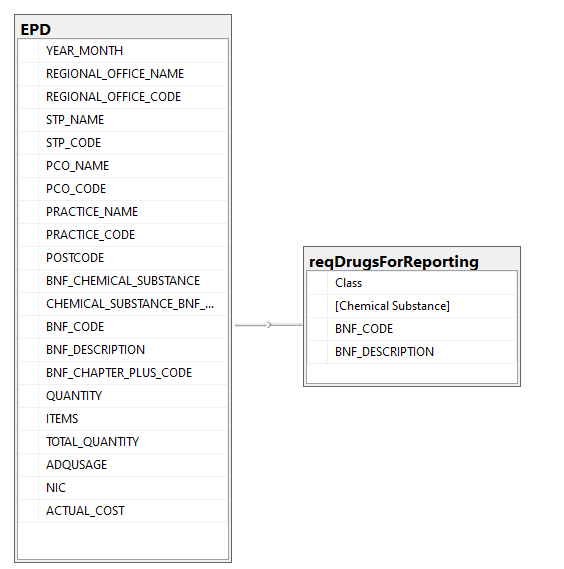


Figure 1 Relationships between data tables.

A step-by-step guide is presented below.

# How To: Import / Export

## Import new data

1. Prepare data file for import:
   1. Monthly Data
      1. Download Monthly EPD CSV
      2. Rename Monthly CSV to :Month.CSV
      3. Place in EPD Directory (F:\EPD\Import)
   2. Required BNF Codes for reporting
      1. Prepare an updated version of the reqDrugsForReporting.csv
      2. Ensure the csv file includes all 4 required columns and all required drugs.
         (Class, Chemical Substance, Bnf_Code, Bnf_Description)
2. Open SSIS Package in Visual Studio 2019
3. Right click Run Package on Monthly EPD Data Import Package or Required Drugs import Package.
   1. Wait until package completes.
   2. The package will import all data correctly.
   3. The package will validate data types and structure prior to import.
4. Data validation
   1. Run data validation script the following is checked:
      1. All automated import validations have run correctly.
      2. Duplication has not occurred
      3. Omissions are not present.
      4. All data is distributed as expected.

## Export Required Drugs list

Filter EPD Database to Required drugs list only

Run TempTable.dtsx

This SSIS package will call a view (EPD_Plus_ReqDrugsList). The Result of the View will be dumped into a table (tempFiltered). A Copy of this data will be saved to a .csv file (Export.csv).

## Automation Packages Names and locations

SSIS Solution: ChangeDataTypes.sln

Monthly EPD Data Import Package: Import_RequiredDrugs.dtsx

Required Drugs import Package: ImportMonth.dtsx

File Location:

Import Directory: C:\EPD\Import

File Names:

Monthly EPD Data file: Import\Month.csv
Required Drugs for reporting: reqDrugsForReporting.csv
